# Primary yolk sac tumor of the retroperitoneum: A case report and review of the literature

**DOI:** 10.3892/ol.2014.2162

**Published:** 2014-05-22

**Authors:** YANG-LONG GUO, YING-LI ZHANG, JIAN-QING ZHU

**Affiliations:** Department of Gynecologic Oncology, Zhejiang Cancer Hospital, Hangzhou, Zhejiang 310022, P.R. China

**Keywords:** yolk sac tumor, primary retroperitoneal, chemotherapy, α-fetoprotein, prognosis

## Abstract

Yolk sac tumor (YST), also known as an endodermal sinus tumor, is a rare malignant germ cell tumor. Primary retroperitoneal YST (PRYST) is extremely rare and, to the best of our knowledge, has only been described in case reports. The histogenesis of PRYST and the appropriate treatment strategy remain unclear due to the rarity of this type of tumor. The present study reports a case of YST in the retroperitoneum. A 19-year-old female presented with abdominal distension and edema of the lower limbs. A computed tomography scan revealed a large, solid mass located in the retroperitoneum. The tumor size was 20×25×30 cm and widespread metastasis was identified during the exploratory laparotomy. The postoperative histopathology report showed a malignant retroperitoneal tumor (although a YST was initially considered). The patient underwent three surgical procedures and 17 cycles of five different chemotherapy regimens. The patient succumbed to cachexia, which was due to tumor recurrence, and liver and spleen metastases 21 months after diagnosis. PRYST may relapse following surgical treatment; however, surgical resection is currently the optimal treatment method. In this case, bleomycin, etoposide and cisplatin; bleomycin, vincristine and cisplatin; and vincristine and cisplatin chemotherapy regimens were effective for the patient with PRYST, although the tumor was not completely resected. α-fetoprotein (AFP) is an important tumor marker for monitoring PRYST recurrence and observation of elevated serum AFP levels during chemotherapy indicates a poor prognosis.

## Introduction

A yolk sac tumor (YST) is a malignant germ cell tumor (MGCT), which typically occurs in the gonads. Extragonadal YST is rare, particularly in the retroperitoneum (1). Due to the fact that this tumor occurs principally in girls and women of childbearing age, the preservation of fertility is particularly important and maintaining reproductive function to the greatest extent has become the primary strategy (2). Prior to the advent of combination chemotherapy, the prognosis for patients with YST was poor, with an ~80–90% mortality rate within two years of diagnosis (3,4). At the end of the 1970s, the prognosis of YST had improved due to the use of novel chemotherapeutic regimens. In the 1990s, the combination of bleomycin, etoposide and cisplatin (BEP) was demonstrated to be highly active against MGCT and became the standard treatment for this type of tumor (5–7). However, the prognosis for YST remains unsatisfactory. Recent studies have demonstrated that the FIGO stage and tumor-reductive surgery strongly affect the prognosis of this disease (8). Other YST prognosis factors remain unclear.

The YST represents a highly malignant germ cell neoplasm in adult cases and is generally characterized by a high serum α-fetoprotein (AFP) level. Serum AFP is one of the hallmarks of YST and facilitates its diagnosis. The serial measurement serum AFP is useful for monitoring its clinical course and response to treatment (9).

Extragonadal germ cell tumors are considered to be a consequence of the mismigration of germ cells along the urogenital ridge during embryogenesis and are estimated to represent ~2–5% of all adult germ-cell malignancies (9). Extragonadal germ cell tumors are normally located in the mediastinum, retroperitoneum and other locations such as the pineal gland or sacrococcygeal area (9). Extragonadal YSTs located in the retroperitoneum are particularly rare (1). Thus, the histogenesis of primary retroperitoneal YST (PRYST) remains controversial and the appropriate treatment is currently unclear. This study presents a case of YST in the retroperitoneum. Patient provided written informed consent.

## Case report

A 19-year-old female presented with abdominal distension and edema in the lower limbs for six months. The patient was admitted to Chunan Chinese Traditional Medical Hospital (Hangzhou, China) and an abdominal computed tomography (CT) scan revealed a solid mass located in the retroperitoneum (tumor size, 20×25×30 cm). A palliative tumor resection was performed during the first exploratory laparotomy on October 28, 2009; the large pelvic mass was not completely removed and widespread metastasis was found. The histopathology report revealed a malignant retroperitoneal tumor (although a YST was initially considered) and two cycles of single-agent mitomycin (10 mg) chemotherapy were performed by intraoperative intraperitoneal and intravenous administration.

**Figure 1:**
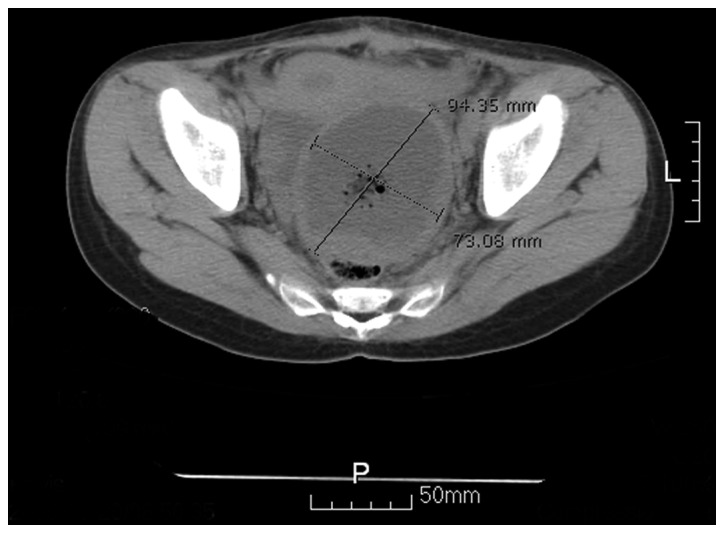
Computed tomography scan of the pelvic mass (size, 9.4×7.3 cm).

The patient was transferred to the Zheijiang Cancer Hospital (Hangzhou, China) on November 17, 2009. The α-fetoprotein (AFP) serum levels were elevated to 9,859.76 ng/ml (normal level, <10 ng/ml); cancer antigen 125 (CA-125) levels were elevated to 51.90 U/ml (normal level, <35 U/ml); the serum β-human chorionic gonadotropin (β-hCG; normal level, <10 mIU/ml), carcinoembryonic antigen (CEA; normal level, <5.0 ng/ml), carbohydrate antigen 19-9 (CA 19-9; normal level, <37 U/ml) and squamous cell carcinoma (SCC) antigen (normal level, <1.5 ng/ml) were within the normal ranges. A pelvic CT scan revealed a pelvic mass (tumor size, 7.4×9.3 cm; Fig. 1) and an upper abdomen CT showed multiple enlargements of the retroperitoneal lymph nodes.

**Figure 2:**
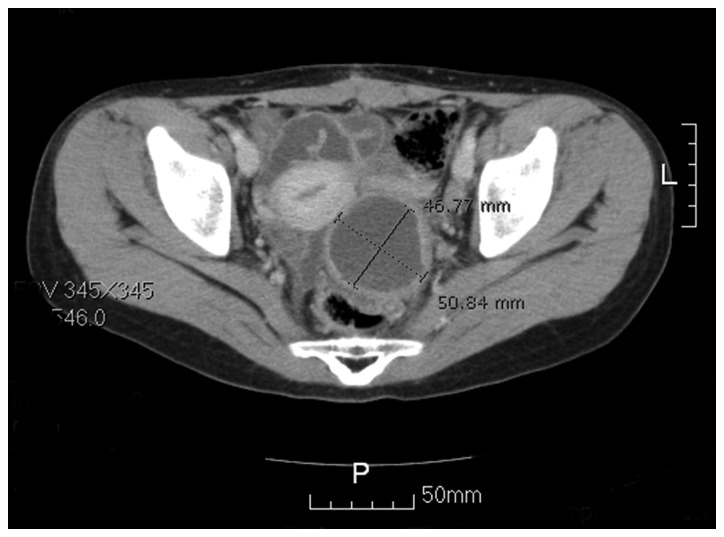
Computed tomography scan of the tumor. The size of the mass was 4.6×5.0 cm, which was significantly reduced compared with Fig. 1.

**Figure 3:**
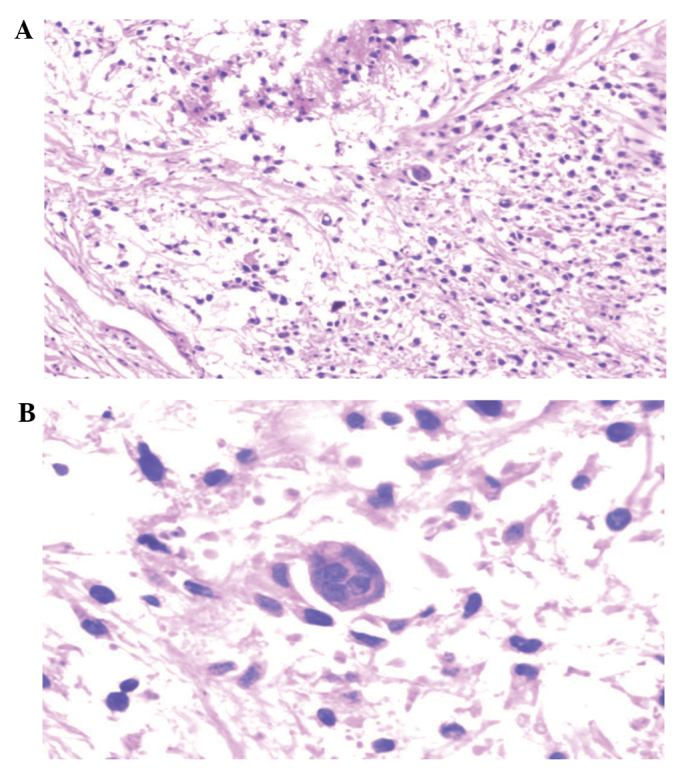
Histopathology revealed a marginal quantity of tumor tissue in the pelvic floor and no positive lymph nodes. (A) Heterotypic cell degeneration (hematoxylin and eosin stain; magnification, ×100) and (B) polykaryocytes (hematoxylin and eosin stain; magnification, ×400) were observed.

Following two cycles of chemotherapy consisting of bleomycin (15 mg for three consecutive days), etoposide (150 mg for four consecutive days) and cisplatin (40 mg for three consecutive days; termed a BEP regimen), the serum AFP levels decreased to 1,251.27 ng/ml on January 7, 2010 and the CT scan revealed that the tumor size had significantly reduced (Fig. 2). The interval debulking and fertility-sparing surgeries (unilateral left side salpingo-oophorectomy, omentectomy and intumescent lymph node resection) were performed on January 12, 2010. No residual tumor was found and the histopathology report showed a marginal quantity of tumor tissue in the pelvic floor and no positive lymph nodes (Fig. 3). Following surgery, an additional two cycles of chemotherapy, consisting of the aforementioned BEP regimen, were administered continuously. Following surgery and an additional two cycles of the BEP regimen, the serum AFP levels decreased to 8.17 ng/ml following the final administration of the BEP regimen on February 28, 2010.

**Figure 4:**
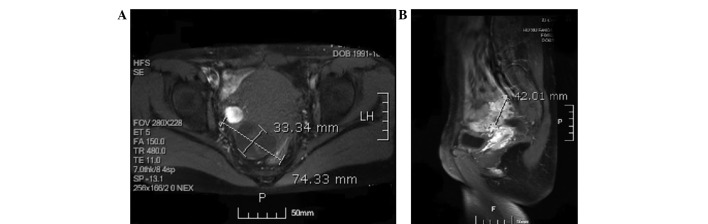
Magnetic resonance imaging revealed a cystic and solid mass in the right parametrium, measuring 3.3×7.4×4.2 cm. The tumor diameter was (A) 3.3cm×7.4 cm in the transverse plane and (B) 4.2cm in the coronal plane.

**Figure 5:**
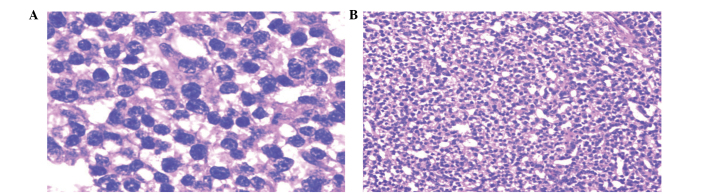
Histopathology report showed a metastatic or invasive malignant tumor (yolk sac tumor was first considered) on the surface of sigmoid colon and the rectum. The cells were (A) round and slightly irregular, with large, circular and hyperchromatic nuclei (hematoxylin and eosin stain; magnification, ×400) and (B) arranged in the solid, glandular cavity (hematoxylin and eosin stain; magnification, ×100)

However, tumor recurrence occurred three months after the final BEP regimen. The serum AFP levels elevated to 193.99 ng/ml on June 4, 2010 and magnetic resonance imaging (MRI) revealed a cystic and solid mass in the right parametrium (Fig. 4). A salpingostomy and secondary cytoreductive surgery were performed on June 11, 2010. No residual tumor was found and the histology report showed metastatic or invasive malignant tumors (although a YST was initially considered) on the surface of the sigmoid colon and the rectum (Fig. 5). The patient underwent three cycles of chemotherapy consisting of bleomycin (15 mg for three consecutive days), vincristine (1.5 mg on day one) and cisplatin (40 mg for three consecutive days; termed a BVP regimen) and three cycles of chemotherapy consisting of vincristine (1.5 mg one day one) and cisplatin (40 mg for three consecutive days; termed a VP regimen). Following the final VP regimen on October 11, 2010, the AFP level decreased to 2.59 ng/ml and the CA-125, β-hCG, CEA, CA 19-9 and SCC were within the normal ranges.

**Figure 6:**
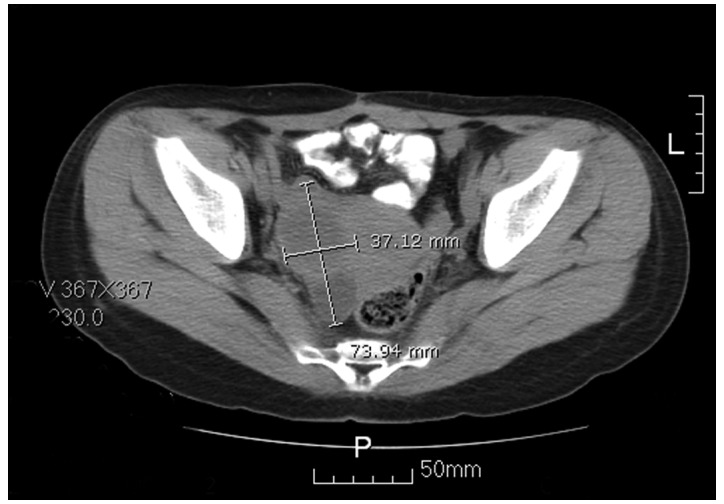
Computed tomography revealed the cystic and solid mass in the right parametrium markedly increased (measuring 3.7×7.3 cm).

**Figure 7:**
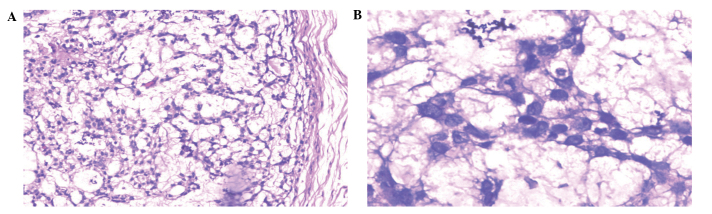
Histopathological analysis demonstrated spindle cells, which were initially considered to be a yolk sac tumor, on the surface of the small intestine. The cells were (A) arranged in a reticular formation of microcapsules and maze-like cracks, with a basophilic myxoid matrix background (hematoxylin and eosin stain; magnification, ×100) and (B) of cubic or flat shape with hyperchromatic nuclei and indistinct nucleoli (hematoxylin and eosin stain; magnification, ×400).

However, tumor recurrence occurred just three months following the final BVP regimen. The serum AFP level elevated to 72.80 ng/ml on January 10, 2011 and the CT scan revealed that the cystic and solid mass in the right parametrium had markedly increased (compared with the prior MRI) (Fig. 6). Radical surgery (hysterectomy, unilateral right side salpingo-oophorectomy and right pelvic lymphadenectomy) was performed on January 18, 2011. No residual tumor was found and the histopathology report showed spindle cells (although a YST was initially considered) on the surface of the small intestine (Fig. 7).

Following surgery, the patient received two cycles of chemotherapy consisting of vincristine (1 mg on day one), actinomycin D (400 μg for five consecutive days) and cyclophosphamide (200 mg for three consecutive days; termed a VAC regimen). However, the serum AFP level increased to 465.27 ng/ml following the final VAC regimen on March 14, 2011 and the positron emission tomography/CT revealed tumor metastases to the liver and the spleen. Therefore, the patient was administered three cycles of chemotherapy consisting of taxol^®^ (210 mg on day one), ifosfamide (2 g on day one and 1 g on days two and three) and cisplatin (70 mg on day one; termed a TIP regimen), while the serum AFP level continuously increased to 3,500.01 ng/ml following the final TIP regimen on May 23, 2011. The patient presented with liver and spleen metastases and succumbed to cachexia 21 months after diagnosis.

## Discussion

PRYST is an extremely rare tumor that, to the best of our knowledge, has only previously been described in case reports. There are no specific clinical symptoms and signs of PRYST; thus, the tumor is commonly identified when it has grown to a considerable size. In the majority of cases, the tumor has invaded the crucial nerves and blood vessels, such as the abdominal aorta, inferior vena cava and may have formed tumor thrombus in the vena cava. It is particularly difficult to manage these cases; however, complete resection of retroperitoneal tumors is crucial for successful treatment. DiPerna *et al* (10) indicated that the resection of tumors, which are invading major vascular structures, may provide an acceptable morbidity and mortality among patients. Maintaining the female reproductive function to the greatest extent has become the primary strategy, during the treatment of gynecological cancer, for prolonging survival and improving patient quality of life. The reproductive function may be retained provided that the uterus and the contralateral ovary remain intact, regardless of the tumor stage (3). Cicin *et al* (2) considered that fertility-sparing surgery was as effective as radical surgery in patients with an ovarian YST. Peccatori *et al* (11) retrospectively analyzed 129 patients with malignant ovarian germ cell tumors and found that fertility-sparing surgery did not affect recurrence or survival rate in patients with ovarian germ cell tumors. Ayhan *et al* (12) analyzed 45 patients with all stages of dysgerminomas and found no significant difference between conservative and non-conservative surgery in recurrence or survival rate of patients. Furthermore, Zanagnolo *et al* (13) reported that fertility-sparing surgery was safe for patients with malignant ovarian germ cell tumors. In the present case, four different surgical procedures, including palliative, interval debulking, fertility-sparing, secondary cytoreductive and radical surgeries, and a salpingostomy, were performed. The serum AFP level markedly decreased following all the procedures except radical surgery. Although the PRYST relapsed, the present study indicated that surgical resection is the optimal treatment modality for PRYSTs, particularly when performed prior to PRYST recurrence.

AFP is an important tumor marker of YST; an increased serum AFP level is typically observed in patients presenting with a YST and exhibits a good correlation with the severity of the lesion. Serum AFP levels decrease rapidly following tumor resection, however, the levels increase during tumor recurrence or metastasis. Talerman *et al* (14) reported that serial serum AFP may be used for diagnostic purposes, and the detection of metastases and recurrence. In the present case, the serum AFP level decreased from 9,859.76 ng/ml to 8.17 ng/ml following interval debulking surgery, fertility-sparing surgery and the BEP chemotherapy regimen; and decreased from 193.99 ng/ml to 2.59 ng/ml following salpingostomy, secondary cytoreductive surgery, and the BVP and VP chemotherapy regimens. By contrast, the serum AFP level increased from 8.17 ng/ml to 193.99 ng/ml following the first tumor recurrence, and from 2.59 ng/ml to 72.80 ng/ml following the second tumor recurrence. Therefore, AFP is an important tumor marker for monitoring tumor recurrence and may be used to assess preoperative or postoperative residual tumors, monitor the response to chemotherapy treatment and contribute to long-term follow-up.

Since the 1980s, various platinum-based chemotherapy regimens (such as BEP and BVP) have been widely used and have markedly improved the prognosis of patients with MGCTs. The administration of cisplatin-based combination chemotherapy regimens has improved the curative effect on MGCT patients. Cicin *et al* (2) reported that the most decisive prognostic factors in patients with ovarian YST were optimal cytoreductive surgery and the standard BEP regimen. In addition, cisplatin-containing chemotherapy has markedly improved the outlook for patients with MGCT and overall cure rates are >80% (15). The BEP regimen became the most effective treatment for MGCTs after the 1990s and is considered to be the first-line chemotherapy regimen for MGCTs (1,15). In the present study, the BEP regimen appeared to be an effective treatment strategy for the PRYST even when the tumor was not completely resected. The serum AFP level markedly decreased (from 9,859.76 ng/ml to 8.17 ng/ml) following four courses of BEP chemotherapy. In addition, the BVP and VP regimens also proved effective following tumor recurrence. The serum AFP level markedly decreased (from 193.99 ng/ml to 2.59 ng/ml) following three cycles of BVP and VP regimens (the BVP regimen was replaced by the VP regimen as the patient had received the life-time dose of bleomycin).

The VAC and TIP regimens are classic chemotherapy regimens for germ cell tumors. Lertkhachonsuk *et al* (16) reported that the VAC regimen was effective for patients with MGCTs. Park *et al* (17) demonstrated that TIP chemotherapy was a well-established and active regimen for patients with relapsed germ cell tumors as a salvage treatment. In the present study, the patient was considered to be a cisplatin-refractory case as, following BEP, BVP and VP regimens, the patient experienced complete remission (defined as no residual tumor and a normal serum AFP level) for approximately three months after each treatment (from February 28 to June 4, 2010 and from October 11, 2010 to January 10, 2011). Thus, as a result of the tumor recurrence, the chemotherapy regimen was altered to the VAC regimen; however, the VAC regimen appeared to be ineffective. Consequently, the chemotherapy regimen was altered again and the TIP regimen was administered which was also ineffective. Accordingly, the platinum-based chemotherapy regimens remain effective for PRYST patients even when there is a tumor relapse following the first platinum-based chemotherapy. By contrast, platinum-based chemotherapy regimens and other regimens may not be effective in the case of a cisplatin-refractory patient where the tumor relapses more than once.

Approximately 10–20% of patients experience a YST relapse following the first treatment (13,18) and their AFP levels may be associated with tumor recurrence and prognosis. Mitchell *et al* (4) reported that relapses were principally observed among patients with an AFP level >1,000 ng/ml. With regards to the prognosis of YST, Mayordomo *et al* (19) reported that a serum AFP level of >1,000 ng/ml was a prognostic factor in patients with ovarian and extragonadal MGCTs. de La Motte Rouge *et al* (20) retrospectively analyzed 84 patients with ovarian YST and found that a decline in the serum AFP level may be a poor prognostic factor. Moreover, high serum AFP levels may be associated with a worse prognosis in patients with MGCTs (4,19); however, these studies that explicitly evaluated the significance of serum AFP levels in an ovarian YST series failed to illustrate that this was a prognostic factor (3,4,8–22). In the present case, the serum AFP level increased from 72.80 ng/ml to 3,500.01 ng/ml during VAC and TIP chemotherapy, which indicated a poor prognosis regardless of radical surgery.

In conclusion, the current study presented a rare case of YST originating in the retroperitoneum. PRYST is an extremely rare malignant tumor with a poor prognosis. Although PRYST may relapse promptly after surgical treatment, surgical resection is considered to be the optimal treatment, particularly when performed prior to PRYST recurrence, as it markedly decreases the AFP level. In addition, it was observed that the BEP, BVP and VP chemotherapy regimens are effective for patients with PRYST even when the tumor is not completely resected. Thus, AFP is an important tumor marker for monitoring PRYST recurrence and the observation of elevated serum AFP levels during chemotherapy indicate a poor prognosis.

## Abbreviations

YST: yolk sac tumor
PRYST: primary retroperitoneal yolk sac tumor
AFP: α-fetoprotein
CT: computed tomography
MRI: magnetic resonance imaging
MGCT: malignant germ-cell tumors
BEP: bleomycin, etoposide and cisplatin
BVP: bleomycin, vincristine and cisplatin
VP: vincristine and cisplatin
VAC: vincristine, actinomycin D and cyclophosphamide
TIP: taxol, ifosfamide and cisplatin
